# Widening the Phenotype of Fragile‐X Tremor Ataxia Syndrome in Females: Spasmodic Dysphonia in Two Patients

**DOI:** 10.1002/mdc3.14061

**Published:** 2024-05-30

**Authors:** Sana Khan, Stefan Williams, Jeremy Cosgrove, John Bamford, Jane Alty

**Affiliations:** ^1^ Sheffield Teaching Hospitals NHS Trust Sheffield UK; ^2^ Leeds Teaching Hospitals NHS Trust Leeds UK; ^3^ College of Health and Medicine, University of Tasmania Hobart Australia

**Keywords:** ataxia, dystonia, FXTAS, premutation, tremor

Fragile‐X tremor ataxia syndrome (FXTAS) is an adult‐onset neurodegenerative disorder caused by a premutation (55‐200 CGG trinucleotide repeats) in the *FMR1* gene on the X‐chromosome. Whilst related to Fragile‐X syndrome (FXS), the latter is caused by a ‘full’ mutation (>200 repeats) in the same gene.^1^ The premutation results in upregulation of gene expression; excessive quantities of the resulting protein is neurotoxic and leads to neurodegeneration in later‐adulthood.

Penetrance is lower in females (16–20%) than in males (40–75%), so most female carriers do not develop FXTAS.^2^ Females have a milder phenotype due to its X‐linked nature and Lyonisation.^1^ FXTAS classically presents with tremor or ataxia in males, who may develop parkinsonism and cognitive decline later.^3^ The female spectrum is wider and includes neuropathy, migraine, seizures, immune‐mediated (hypothyroidism) and vestibular disorders, and (Fragile‐X associated) premature ovarian insufficiency.^4^ The mechanisms that govern these differences are uncertain.

It has previously been suggested that female *FMR1* premutation carriers may present with dystonia^5^ (e.g., cervical^6^ and oromandibular^7^); the cortico‐ponto‐cerebello‐thalamo‐cortical pathway has been implicated in the pathophysiology of dystonia, possibly explaining this association.^8^

Laryngeal dystonia, or spasmodic dysphonia (SD), is a focal dystonia of the vocal cords that usually affects middle‐aged/elderly females and results in involuntary spasms of laryngeal musculature. There are two types: adductor and abductor, which result in a strangulated/croaky and breathy voice, respectively.^9^ Horvath et al^10^ first described SD as a presenting feature of female FXTAS in a 60‐year‐old female with a postural upper‐limb and vocal tremor who developed ataxia 8 years later. Here, we describe two females who both had SD as part of their FXTAS spectrum; to the best of our knowledge, this is only the second such report. We propose that FXTAS should be considered in females who present with dystonic vocal changes, which may present many years before tremor or ataxia.

The first case is an 83‐year‐old female who presented with “croaky” voice changes 25 years ago. She was declined a job in a call center due to concerns that her voice may be unsettling. She was assessed by a neurologist (JB) and ear, nose and throat specialist and diagnosed with SD. Vocal exercises did not lead to improvement. She declined laryngeal botulinum toxin injections.

Seven years later, she developed a mild head‐and bilateral upper‐limb action tremor which reduced in severity with alcohol. She was diagnosed with essential tremor (ET). At a similar time, the son of her sister's daughter was diagnosed with FXS. This triggered genetic testing in the family and led to her *FMR1* premutation (>2.8 + 5.2 kb bands on Southern blot testing) being discovered.

After a further 7 years, she developed an ataxic gait. She was referred to the national ataxia center for an opinion on whether her symptoms could all be explained by her premutation. A history of developmental delay and learning difficulties in her adult son coupled with the wide and variable female phenotype raised suspicion of FXTAS. An MRI brain scan was arranged, which showed mild–moderate atrophy of her cerebellar vermis and superior cerebellar hemispheres, compatible with a milder female presentation.

Three years later, she developed bilateral foot drop and reduced sensation below her knees. Nerve conduction tests confirmed a mild–moderate length‐dependent sensorimotor axonal neuropathy.

The second case is a 79‐year‐old female with a history of premature menopause (aged 38). She presented 10 years ago with a mild, irregular head tremor (right torticollis, responding to *geste antagoniste*) and left‐sided upper‐limb postural and intention tremor. Lower‐limb reflexes were reduced, but there was no ataxia. She had altered, breathy speech. A CT brain scan was arranged to rule out a cerebellar abnormality, but only showed age‐related cerebral atrophy. She was diagnosed with ET.

Nine months later, she developed an ataxic gait and vocal tremor, with worsening of her head tremor. At a similar time, her son was diagnosed with FXS which prompted genetic testing in her family; subsequently, her *FMR1* premutation was found (somatic mosaic of 98–105 repeats on PCR testing). Three years later, she was found to have reduced vibration but preserved light‐touch sensation below her knees bilaterally. An MRI brain scan only showed age‐related involutional changes. Botulinum toxin injections were commenced for her head tremor.

Four years later, she struggled to project her voice, resulting in speech therapy input. Flexible endoscopy demonstrated a pharyngeal tongue‐based tremor and simultaneous supraglottic tremor consistent with SD. Vocal cord exercises led to minimal improvement (Audio 1).

**Audio 1:** Audio recording conversation of case two with a neurologist (SW, co‐author).

It has previously been suggested that dystonia should be included within the FXTAS spectrum, as it is not currently part of diagnostic criteria.^5^ Clinicians should consider FXTAS in females who present with vocal tremors or SD as the phenotype may be wider than has previously been appreciated. Whilst SD and tremor may co‐occur in idiopathic dystonia, ataxia and neuropathy are clues that suggest FXTAS.

## Author Roles

(1) Idea: A. Conception of idea; (2) Manuscript preparation: A. First draft B. Revisions and critique C. Approval of final manuscript; (3) Patient consent: A. Obtaining informed consent.

S.K.: 2A, 2B, 2C, 3A

S.W.: 2A, 2B, 2C, 3A

J.C.: 2B, 2C

J.B.: 1A, 2B, 2C

J.A.: 1A, 2B, 2C, 3A

## Disclosures

**Ethical compliance statement:** This case report did not require use of an institutional review board or ethics committee. Informed consent was obtained verbally (in person and via telephone) and in writing (remotely/in person). We confirm that we have read the Journal's position on issues involved in ethical publication and affirm that this work is consistent with those guidelines.

**Funding sources and conflicts of interest:** No specific funding was received for this work. The authors declare that there are no conflicts of interest relevant to this work.

**Financial disclosures for the previous 12 months:** The authors declare that there are no additional disclosures to report.
